# Fermentation of Lupin Protein Hydrolysates—Effects on Their Functional Properties, Sensory Profile and the Allergenic Potential of the Major Lupin Allergen Lup an 1

**DOI:** 10.3390/foods10020281

**Published:** 2021-01-31

**Authors:** Katharina Schlegel, Norbert Lidzba, Elke Ueberham, Peter Eisner, Ute Schweiggert-Weisz

**Affiliations:** 1 Department of Chemistry and Pharmacy, Friedrich-Alexander-Universität Erlangen-Nürnberg, 91054 Erlangen, Germany; katharina.schlegel@ivv.fraunhofer.de; 2 Department Food Process Development, Fraunhofer Institute for Process Engineering and Packaging (IVV), 85354 Freising, Germany; peter.eisner@ivv.fraunhofer.de; 3 Department of Therapy Validation, Fraunhofer Institute for Cell Therapy and Immunology (IZI), 04103 Leipzig, Germany; norbert.lidzba@izi.fraunhofer.de (N.L.); elke.ueberham@izi.fraunhofer.de (E.U.); 4 ZIEL-Institute for Food & Health, TUM School of Life Sciences Weihenstephan, Technical University of Munich, 85354 Freising, Germany; 5 Faculty of Technology and Engineering, Steinbeis-Hochschule, 01069 Dresden, Germany; 6 Institute of Nutritional and Food Sciences, University of Bonn, 53115 Bonn, Germany

**Keywords:** enzymatic hydrolysis, fermentation, lupin protein, functional properties, sensory profile, lupin allergy, lup an 1, plant protein

## Abstract

Lupin protein isolate was treated using the combination of enzymatic hydrolysis (Papain, Alcalase 2.4 L and Pepsin) and lactic acid fermentation (*Lactobacillus sakei* ssp. *carnosus*, *Lactobacillus amylolyticus* and *Lactobacillus helveticus*) to investigate the effect on functional properties, sensory profile and protein integrity. The results showed increased foaming activity (2466–3481%) and solubility at pH 4.0 (19.7–36.7%) of all fermented hydrolysates compared to the untreated lupin protein isolate with 1613% of foaming activity and a solubility of 7.3 (pH 4.0). Results of the SDS-PAGE and Bead-Assay showed that the combination of enzymatic hydrolysis and fermentation of LPI was effective in reducing *L. angustifolius* major allergen Lup an 1 to a residual level of <0.5%. The combination of enzymatic hydrolysis and fermentation enables the production of food ingredients with good functional properties in terms of protein solubility and foam formation, with a balanced aroma and taste profile.

## 1. Introduction

The global population is expected to grow by 2 billion to 9.7 billion people over next 30 years [1]. In order to maintain the world’s population with protein, food systems will be faced with a major challenge. An increase in animal production would not be a sustainable option to meet the high demand of protein. Supplying the world with animal protein has drastic effects on the environment, which include the intensive use of land, the deterioration of air and water quality and the emission of greenhouse gases. [2]. A promising way to reduce the impact of nutrition on the environment could be the partial replacement of animal proteins with plant proteins. Legumes such as lupins are becoming more and more popular as an alternative source of protein to animal protein and soy. Lupins are widely cultivated in Europe and in South America and are particularly attractive for human consumption because of their high protein content of 39%, up to 55% (dry matter) [3] with a well-balanced amino acid profile and a low carbohydrate content compared to other legumes [4]. However, the use of lupin proteins in some foods like refreshing drinks is limited due to the characteristics of their functional properties, in particular their solubility in the acidic range. Several studies have shown that enzymatic hydrolysis can significantly improve the functional properties of plant proteins such as protein solubility, foaming and emulsification [5,6,7,8,9,10]. In addition, it was shown that the allergenicity of the proteins can be reduced by enzymatic treatments [11,12]. However, protein hydrolysis can also lead to negative modifications of the sensory profile by producing a bitter taste that inhibits their use in food [6,7,12]. One promising approach to influence the sensory profile of these ingredients could be lactic acid fermentation. Several studies have shown that fermentation of plant proteins by lactic acid leads to reduced or masked off-flavors in legumes and improves their sensory profile [13,14,15]. However, lactic acid fermentation is less effective in improving the functional properties of proteins and the degradation of polypeptides to reduce the allergenic potential of those proteins is less effective compared to enzymatic hydrolysis. The combination of enzymatic hydrolysis and fermentation could use the positive effects of both treatments to develop low-allergen food ingredients with excellent functional properties and a balanced sensory profile. The objective of this study was to investigate the effect of the combination of enzymatic hydrolysis and fermentation on functional properties of lupin proteins—protein solubility, foaming properties and emulsification capacity. In addition, the sensory profile of the treated ingredients was also evaluated. In order to obtain first insights of the reduction of the allergenic potential of lupin proteins, both molecular weight distribution and immunological detectability of the fermented hydrolysates were compared with untreated lupin protein isolates.

## 2. Materials and Methods

### 2.1. Raw Materials and Chemicals

#### 2.1.1. Lupin Seeds

Lupin (*Lupinus angustifolius* L. cultivar Boregine) seeds were provided by Saatzucht Steinach GmbH & Co KG (Steinach, Germany).

#### 2.1.2. Enzymes

The sources, types and supplier of the enzymes used in this study are listed in Table 1. Proteolytic enzyme preparations were chosen according to a previous study [7], in which promising results were achieved by those enzyme preparations in lupin protein degradation.

#### 2.1.3. Strain Selection

The fermentation of lupin protein hydrolysates was carried out using *Lactobacillus sakei* ssp. *carnosus* (DSM 15831), *Lactobacillus amylolyticus* (TL 5) and *Lactobacillus helveticus* (DSM 20075). Microorganisms were purchased from Deutsche Sammlung von Mikroorganismen und Zellkulturen (Braunschweig, Germany) and Chair of Brewing and Beverage Technology (Technical University Munich, Germany). The microorganisms were stored as a cryoculture in our strain collection and were activated on MRS (De Man, Rogosa & Sharpe) agar. The selection of the microorganisms were chosen according to Schlegel, Leidigkeit, Eisner and Schweiggert-Weisz [15], based on the promising results achieved in the aroma formation and hedonic evaluation.

#### 2.1.4. Nutrient Media

Liquid growth media and agar were obtained from Carl Roth (Karlsruhe, Germany).

### 2.2. Preparation of Lupin Protein Isolate

Lupin protein isolate (LPI) was prepared from *Lupinus angustifolius* L. cultivar Boregine. Lupin seeds were dehulled, separated and passed through a roller mill. The resulting flakes were de-oiled in *n*-hexane. Flakes were extracted with 0.5 M HCl (1:8 *w*/*w*) for 1 h. Suspension was separated using a decanter centrifuge at 5600× *g* and 4 °C for 1 h and the supernatant was discarded. The acid pre-extracted flakes were dispersed in 0.5 M NaOH (1:8 *w*/*w*, pH 8.0) for 1 h at room temperature while stirring and separated by centrifugation (5600× *g*, 4 °C, 1 h). The supernatant was adjusted to pH 4.5 with 0.5 M HCl. The precipitated proteins were separated by centrifugation (5600× *g*, 130 min) and neutralized with 0.5 M NaOH, pasteurized at 70 °C for 10 min) and spray-dried using an Anhydro spray dryer (SPX Flow Technology, Charlotte, NC, USA) with an inlet temperature of 180 °C and an outlet temperature of 80 °C at a mass flow rate of 24 kg/h.

### 2.3. Enzymatic Hydrolysis of LPI

Enzymatic hydrolysis of LPI was performed in a 5 L thermostatically controlled reaction vessel, as previously described [7]. Briefly, the protein isolate was dispersed in deionized water with an Ultra-Turrax at 5000 rpm for 1 min (IKA-Werke GmbH & Co. KG, Staufen, Germany) to achieve a protein concentration of 50 g/kg. Temperatures and pH values were adjusted, and enzyme preparation was added (Table 1). The suspension was incubated at controlled pH and temperature (Table 2) with continuous stirring for 2 h. The reaction was stopped by heating up to 90 °C for 20 min, afterwards, the suspension was cooled down to room temperature and neutralized (pH 7) with 1 M NaOH or 1 M HCl. 

### 2.4. Fermentation of Hydrolysed LPI

Hydrolysed LPI was fermented in a 5 L glass reaction vessel in an incubator under aerobic conditions and in a 5 L glass reaction vessel with a bioreactor (Biostat B, Sartorius AG, Goettingen, Germany) under anaerobic conditions, respectively, as described previously [15]. Briefly, 0.5% glucose (*w*/*w*) was added to the 5% hydrolyzed LPI (*w*/*w*) solution, pasteurized at 80 °C for 20 min and inoculated with the activated culture of 10^7^ CFU/mL. Anaerobic conditions for *Lactobacillus helveticus* were achieved by flushing the reactor with N_2_. LPI was fermented at 37 °C (*Lactobacillus sakei* ssp. *carnosus* and *Lactobacillus helveticus*) and 42 °C (*Lactobacillus amylolyticus*), respectively, for 24 h without stirring. Viable cell counts were determined after 0 h and 24 h of fermentation and pH course were recorded for 24 h fermentation with one measurement point at each 30 min (wtw pH 3310 pH electrode, Xylem Analytics Germany GmbH, Weilheim, Germany). The process was stopped by heating the suspension up to 90 °C for 20 min. All samples were neutralized (pH 7.0) with 1 M NaOH and spray dried with a Niro Atomizer 2238 (GEA, Düsseldorf, Germany).

### 2.5. Chemical Composition

Protein content was determined according to the Dumas combustion method AOAC 968.06 (TruMac N, Leco Instruments, Mönchengladbach Germany) using a protein calculation factor of N × 5.8 [16]. The dry matter (105 °C) and ash (950 °C) contents were analyzed according to AOAC methods 925.10 and 923.03 in a TGA 601 thermogravimetric system (Leco Instruments GmbH) at 105 °C and 950 °C, respectively.

### 2.6. Molecular Weight Distribution

The molecular weight distribution of the untreated LPI and fermented LPI hydrolysates was determined by sodium dodecyl sulfate–polyacrylamide gel electrophoresis (SDS-PAGE) as described by Laemmli [17] with modifications [15]. Briefly, LPI and fermented LPI hydrolysates were resuspended in loading buffer (0.125 mol/L Tris-HCl, 4% SDS (*w*/*v*), 20% glycerol (*v*/*v*), 0.2 mol/L DDT, 0.02% bromophenol blue, pH 6.8), dissolved in an ultrasonic bath (30 °C, 30 min), boiled at 95 °C for 5 min (Eppendorf Thermomixer, Eppendorf AG, Hamburg, Germany) and separated with a Mini Spin centrifuge at 12,045× *g* for 10 min (Eppendorf AG). Supernatant was mixed in a ratio of 1:10 with loading buffer (see above). An aliquot of 10 µL of each sample was transferred into the wells of Bio-Rad 4–20% Criterion TGX Stain-Free precast gels (Bio-Rad Laboratories GmbH, Feldkirchen, Germany). The Precision Plus Protein™ Unstained Protein Standard (Bio-Rad Laboratories) was used as molecular weight marker (10–250 kDa). Gels were run at room temperature for 38 min at 200 V (60 mA, 100 W) in a vertical electrophoresis cell (Bio-Rad Laboratories). Protein bands were visualized using a Gel Doc™ EZ Imager system (Bio-Rad Laboratories) and determined using Image Lab software (Bio-Rad Laboratories).

### 2.7. Determination of Lup an 1 with Specific Monoclonal Antibodies

Amount of Lup an 1 was measured by an in house sandwich assay using β-conglutin specific antibodies (Izimab Lup an 1-1 and Izimab Lup an 1-2 (unpublished)) on FLEXMAP 3D^®^ flow analyzer system from Luminex Corporation (Austin, TX, USA). The monoclonal capture antibody, Izimab Lup an 1-1, was coupled onto MagPlex^®^ beads and the monoclonal detection antibody Izimab Lup an 1-2 was biotinylated according to standard procedures. Lupin samples were extracted using denaturing conditions [6]. The samples were incubated with bead conjugated Izimab Lup an 1-1, washed three times in PBS with 0.05% Tween^®^ 20 (PBS-T) and incubated with biotinylated Izimab Lup an 1-2 followed by another wash with PBS-T. Streptavidin-conjugated phycoerythrin (SA-PE) was diluted 1:2000 in assay buffer, 100 µL per well were added to the sandwich complex and agitated for one hour. The reaction was terminated by washing the wells three times with wash buffer and subsequently filled up with 120 µL assay buffer. Readings on FLEXMAP 3D^®^ flow analyzer expressed as median fluorescence intensity (MFI) per 100 beads calculated on the basis of a calibration curve were converted to relative reactivity in percent by dividing the individual MFI by the MFI of the control sample, which represents the LPI used as raw material in the processing steps.

### 2.8. Technofunctional Properties

#### 2.8.1. Protein Solubility

The solubility (%) of LPI and fermented LPI hydrolysates was determined in duplicate at pH 4.0 and 7.0 according to Morr, et al. [18]. The sample was suspended in 0.1 M NaCl (3% *w*/*w*), pH was adjusted (0.1 M HCl) and stirred for 1 h at room temperature. Undissolved fractions of the samples were removed by centrifugation (20,000× *g*, 15 min) at room temperature. The supernatants were filtered with a Whatman No. 1 filter paper. Protein content was determined by a method of Lowry, et al. [19] using the DC Protein Assay (Bio-Rad Laboratories) with a BSA standard curve for calculating the protein concentration. The absorbance was read at 750 nm. The resulting protein content was related to the total amount of protein and protein solubility (%) was determined.

#### 2.8.2. Foaming Properties 

Foaming activity and stability were determined according to Phillips, et al. [20] in duplicate. For foaming activity, a 100 mL solution of 5% protein (pH 4.0 and 7.0) was whipped at room temperature for 8 min in a whipping machine (Hobart 50-N, Hobart GmbH, Offenburg, Germany. The increase of foam volume was defined as foaming activity (%); the percentage of foam volume remaining after 1 h was defined as foam stability (%).

#### 2.8.3. Emulsifying Capacity

Emulsifying capacity was determined at pH 4.0 and 7.0 according to Wang and Johnson [21]. Samples were suspended in deionized water (1% *w*/*w*), adjusted to pH and stirred with an Ultraturrax (IKA-Werke GmbH & Co. KG) at 18 °C. Rapeseed oil was continuously added (10 mL/min) until phase inversion was detected (<10 μS/cm). The volume of added oil was used to calculate emulsifying capacity (mL oil per g sample). Measurements were performed in duplicate.

### 2.9. Sensory Analysis of Fermented Hydrolysates

#### 2.9.1. Panelists

Panelists were trained assessors recruited from Fraunhofer IVV (Freising, Germany), with no known illness and normal olfactory function during the test. The panel consisted of 10 panelists. All panelists were tested for their olfactory function in weekly training sessions with selected suprathreshold aroma solutions. The samples were evaluated during two sessions on one day.

#### 2.9.2. Descriptive Analysis

Samples (2% *w*/*w*) of LPI and fermented LPI hydrolysates were prepared in tap water by stirring. All samples were presented to the panel in covered glass vessels. The panelists were requested to open the lid of the vessels and record the retronasal aroma and taste attributes. After a short discussion, common retronasal aroma attributes with corresponding references and taste attributes were collected and rated on a scale from 0 (no perception) to 10 (strong perception) by each panelist in a separate session. The following ten aroma and taste qualities and corresponding references (given in brackets) were selected by the trained panelists (n = 10) for LPI and fermented LPI hydrolysates: oatmeal-like (oatmeal); cocoa-like (cocoa); malty (methylpropanal); green, grassy (hexanal) pea-like (2–isopropyl-3-methoxypyrazine); fatty ((E,Z)-2,4-nonadienal); cardboard-like, cucumber-like ((E)-2-nonenal); roasty (2-acetylpyrazine); cooked potato-like (3-(methylthio)propanal); earthy (2,3- diethyl-5-methylpyrazine), bitter, salty, sour. The sensory evaluation was carried out once.

### 2.10. Statistical Analysis

Results are expressed as means ± standard deviations and, for sensory evaluation, (aroma profile) as median ± standard deviations. Data were analyzed using pairwise *t*-test to determine the significance of differences between a sample and the untreated LPI, with a threshold of *p* < 0.05. Statistical analysis and visualization were performed with Origin 2018 for Windows (Origin Lab Corporation, Northampton, MA, USA). The results of the sensory evaluation were evaluated using Principal Component Analysis (PCA) covariance matrix to assess aroma and taste qualities. PCA was performed using Origin 2018 for Windows (Origin Lab Corporation).

## 3. Results and Discussion

### 3.1. Chemical Properties

Dry matter content (%) of all fermented LPI hydrolysates were within the range of 92.8% for the Alcalase 2.4 L hydrolysate S4 (*Lactobacillus sakei* ssp. *carnosus*) to 94.0% for the Pepsin hydrolysate S7 (*Lactobacillus sakei* ssp. *carnosus*)—a significantly (*p* < 0.05) lower dry matter content compared to untreated LPI (95.4%) (Table 3).

Untreated LPI contained the highest protein content with 89.6%. Protein content of fermented LPI hydrolysates ranged from 66.8% for *Lactobacillus amylolyticus* fermented Papain hydrolysate (S3) to 78.7% for *Lactobacillus helveticus* fermented Papain hydrolysate (S2).

Ash content (%) was within the range of 4.2% for LPI to 8.8% for the Pepsin hydrolysate S9 (*Lactobacillus helveticus*). The increased ash content of treated samples compared to untreated LPI might be attributed to the addition on NaOH during neutralization after fermentation. 

### 3.2. Comparison of Microbial Growth on Lupin Protein Isolate Solutions

The growing parameters (CFU and pH) for all experiments after 0 h and 24 h of fermentation are shown in Table 4 and Table 5. The results showed that all microorganisms were able to grow in hydrolyzed LPI. The minimum increase in CFU/mL (ΔE_CFU/mL_) was recorded for Pepsin hydrolysate S9 (*Lactobacillus helveticus*) with 1.02 × 10^8^ CFU/mL and the maximum for Pepsin hydrolysate S8 (*Lactobacillus amylolyticus*) with 1.32 × 10^9^ CFU/mL. 

**Figure 1:**
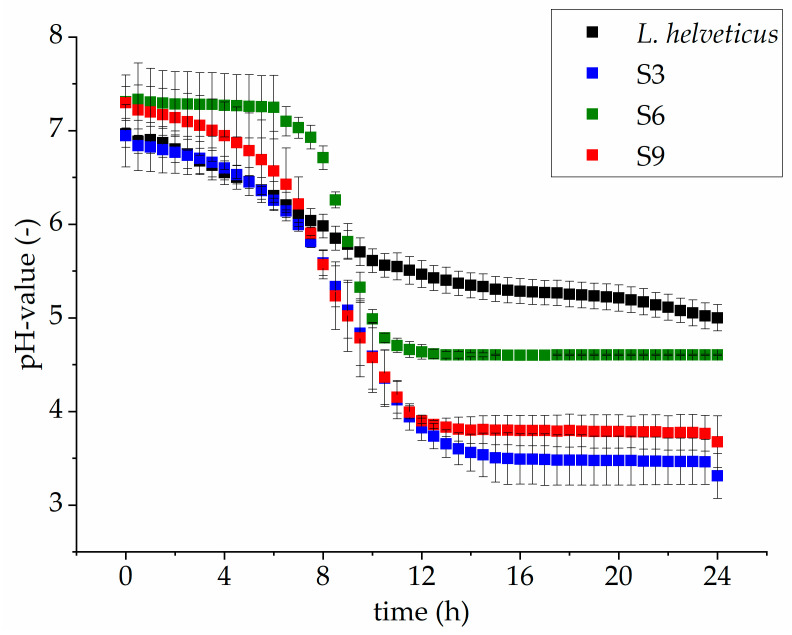
Course of pH value for *Lactobacillus helveticus* fermentation on LPI (black curve) and on Papain (S3, blue curve), Alcalase 2.4 L (S6, green curve) and Pepsin (S9, red curve) LPI hydrolysates over 24 h fermentation. The data are expressed as mean ± standard deviations from duplicates.

The lowest pH values after 24 h of fermentation were recorded for Papain hydrolysate S3 (*Lactobacillus helveticus*) and Pepsin hydrolysate S9 (*Lactobacillus helveticus*) with pH values of 3.3 and 3.7, respectively. Fermentation of hydrolysates obtained by Alcalase 2.4 treatment (S4–S6) tended to show higher pH values compared to hydrolysates obtained by other proteolytic enzyme preparations. Presumably, Alcalase 2.4 degradation leads to a higher buffer capacity of the respective samples. The lowest pH reduction (5.1) was achieved by Alcalase 2.4 hydrolysate S4 (*Lactobacillus sakei* ssp. *carnosus*). A former study described a rapid decrease in pH for *Lactobacillus helveticus* and *Lactobacillus amylolyticus* during the fermentation of LPI [15]. In this work, the pH curve over 24 h of fermentation was also recorded (Figure 1 shows exemplarily the pH curve for LPI samples (not hydrolyzed) fermented with *Lactobacillus helveticus*. It was observed that *Lactobacillus helveticus* showed an extended lag phase of 7 h in decreasing the pH value than *Lactobacillus helveticus* did on Papain (S3) and Pepsin (S9) hydrolysate, respectively. However, after 24 h of fermentation, *Lactobacillus helveticus* was able to decrease the pH in the range of 3.1 and 4.6 in all hydrolysates. 

### 3.3. Molecular Weight Distribution (SDS-PAGE) and Immunoreactivity

**Figure 2:**
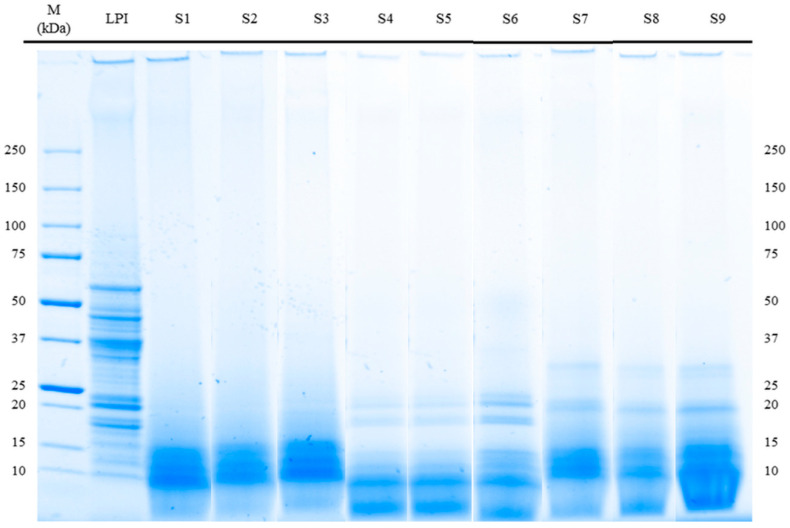
Peptide band profiles of LPI and fermented LPI hydrolysates as determined by SDS-PAGE under reducing conditions.

**Figure 3:**
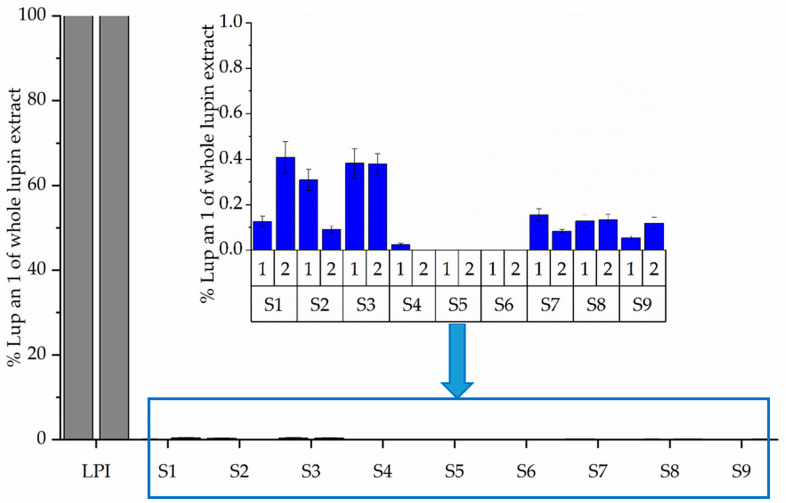
Determination of Lup an 1 with specific monoclonal antibodies (Izimab Lup an 1-1 and Izimab Lup an 1-2) of unfermented LPI and fermented LPI hydrolysates using Bead-Assay. Results are shown as mean ± standard derivation of each duplicate (1 and 2). Each Bead-Assay was performed in triplicate.

The molecular weight distribution (SDS-PAGE) of LPI and its fermented hydrolysates was used to determine the protein integrity and is shown in Figure 2. All treatments resulted in prominent changes in the SDS-PAGE profile with hydrolyzed polypeptides to smaller fragments with molecular weights below 30 kDa. Enzymatic hydrolysis seems to have the greatest influence on the degradation of polypeptides of LPI. Comparing the profiles of the molecular weight distribution of a previous study [7] after hydrolysis of LPI, it is shown that the profiles did not change visibly compared to those after the combination of hydrolysis and fermentation. This observation is also supported by the results of a further study [15], which show that fermentation has only minor influence on the profiles of molecular weight distribution. Furthermore, it is described that β-conglutin with a molecular weight of ~55–61 kDa is known as the major allergen of *L. angustifolius* L. (Lup an 1) [22]. The SDS-PAGE results of all fermented LPI hydrolysates showed a degradation of the described IgE-reactive polypeptide (Figure 2) independent of the enzyme preparations used. These observations were confirmed by the results of the Bead-Assay (Figure 3). A significant reduction in the signal intensity of Lup an 1 below 0.5% was observed for all fermented hydrolysates compared to unfermented LPI (100%) due to the combination of enzymatic hydrolysis and fermentation The highest decrease of immunological reactivity was achieved by treatment with Alcalase 2.4 L combined with fermentation S5 (*Lactobacillus amylolyticus*) and S6 (*Lactobacillus. helveticus*). No binding of Lup an 1 antibodies in sandwich format could be detected (read out not detectable). The assumption that enzymatic hydrolysis is a powerful approach to reduce allergenic potential is supported by further studies [23,24,25,26,27].

### 3.4. Technofunctional Properties

#### 3.4.1. Protein Solubility

Protein solubility of LPI and fermented LPI hydrolysates was determined as a function of pH at pH 4.0 and pH 7.0 and is given in Table 6. All samples showed higher protein solubility at pH 7.0 than at pH 4.0. Solubility decreases as pH value approaches the isoelectric point, approximately pH 4.5–5.0, as discussed frequently [4,7,12,28,29]. Protein solubility at pH 7.0 ranged for all samples, from 45.2% for the Papain hydrolysate S2 (*Lactobacillus helveticus*) to 66.6% for Papain hydrolysate S3 (*Lactobacillus amylolyticus*). However, a significant difference between LPI and the fermented hydrolysates (with the exception of Pepsin hydrolysate S6 (*Lactobacillus helveticus*)) could not be observed.

In contrast, protein solubility at pH 4.0 after enzymatic and fermentation treatment was significantly different (*p* < 0.05) in comparison to untreated LPI (7.3%). All fermented hydrolysates showed significant higher protein solubility with values between 19.7% and 36.7%. The minimum protein solubility (19.7%) of the fermented hydrolysates was determined after fermentation of the Papain hydrolysate S2 (*Lactobacillus helveticus*). The fermentation of a nonhydrolyzed lupin protein isolate with *Lactobacillus helveticus* also resulted in very low protein solubility at pH 4.0 and 7.0 in a former study [15] The single hydrolysis of LPI by means of Alcalase 2.4 L resulted in a considerable increase in protein solubility compared to the hydrolysis by means of other proteolytic enzyme preparation [7], confirming high proteolytic activity of the Alcalase 2.4 L preparation. However, the tendency for the highest protein solubility for Alcalase 2.4 L-hydrolyzed LPI at both pH values and significantly lower protein solubility for Papain and Pepsin-hydrolyzed LPI compared to Alcalase 2.4 L hydrolysate was not observed in this study. A significant difference between the applied enzymes could not be identified.

#### 3.4.2. Foaming Properties

Foaming activity (%) and foam stability (%) of untreated LPI and fermented LPI hydrolysates are given in Table 6. All treated samples showed significantly (*p* < 0.05) higher foaming activity, with values from 1819% up to 2789% at pH 4.0 and 2466% up to 3481% at pH 7.0, compared to untreated LPI with values of 828% and 1613%, respectively. The highest activities with values of 3338%, 3443% and 3481% were determined for Pepsin hydrolysates (S7–S9) at pH 7.0. A high foaming activity of 3614% was also observed at pH 7.0 after single hydrolysis of LPI with Pepsin [7]. The foaming activities at pH 4 were lower than those at pH 7 for all samples, with the exception of the samples hydrolyzed with Alcalase 2.4 L (S4–S6). It is assumed that the lower foam activity under acidic conditions was caused by the significantly lower protein solubility of the samples at pH 4.0 in comparison to pH 7.0.

Furthermore, previous studies have shown that foaming activity of LPI increases only slightly through fermentation [15,30]. Therefore, enzymatic treatment seems to have the greatest effect on foaming activity in this study. Enzymatic hydrolysis breaks larger polypeptides into smaller peptides, thus improving foam formation by rapid diffusion at the air–water interface [31]. Furthermore, Meinlschmidt, Schweiggert-Weisz and Eisner [26] have shown an increase in foaming activity of soy protein isolates after enzymatic hydrolysis and fermentation.

LPI showed foam stability (%) of 89% at pH 7.0 after 1 h standing. The samples treated with *Lactobacillus amylolyticus* and Pepsin (S8), as well as the samples treated with *Lactobacillus sakei* ssp. *carnosus* and Alcalase 2.4 L (S4), did not show significant differences in foam stability with 90% and 96%, respectively, in comparison to the untreated LPI. All other treated samples showed significantly lower or even no foam stability. At pH 4.0, the untreated LPI showed foam stability of 92%. All fermented LPI hydrolysates did not show any foam stability. Foams can be stabilized by large peptides with flexible structures. Hydrolysis reduces protein surface coverage, which means that the air–water interface is no longer stabilized and foam collapse occurs in the hydrolyzed protein foams [32].

#### 3.4.3. Emulsifying Capacity

The emulsifying capacity of untreated LPI at pH 7.0 was 666 mL/g. As shown in Table 6, all treated samples showed significantly (*p* < 0.05) lower emulsifying properties than LPI with values lower than 477 mL/g. The samples hydrolyzed with Alcalase 2.4 L showed the lowest emulsifying properties with values of 246 (S4), 283 (S5) and 323 mL/g (S6). The emulsifying capacity at pH 4.0 showed lower values than at pH 7.0 for all samples, with the exception of the samples treated with Alcalase 2.4 L (S4–S6). El-Adawy, Rahma, El-Bedawey and Gafar [32] found a direct correlation between the emulsifying properties and solubility of a protein. In this study, we could not find a correlation between the solubility of proteins and their ability to form an emulsion within the same pH value. However, we observed that emulsification capacities were lower at pH 4.0 than at pH 7.0. In addition, the samples at pH 4.0 showed lower protein solubilities than at pH 7.0. 

### 3.5. Sensory Analysis

Fermentation enables changes in sensory profiling through the generation and degradation of flavor active compounds. Proteins, carbohydrates and fats from the raw materials provide the necessary precursors, e.g., for the formation of volatile aroma-active compounds. Most of the formation pathways of aroma-active and organic compounds are based on functional metabolic pathways of lactic acid bacteria.

**Figure 4:**
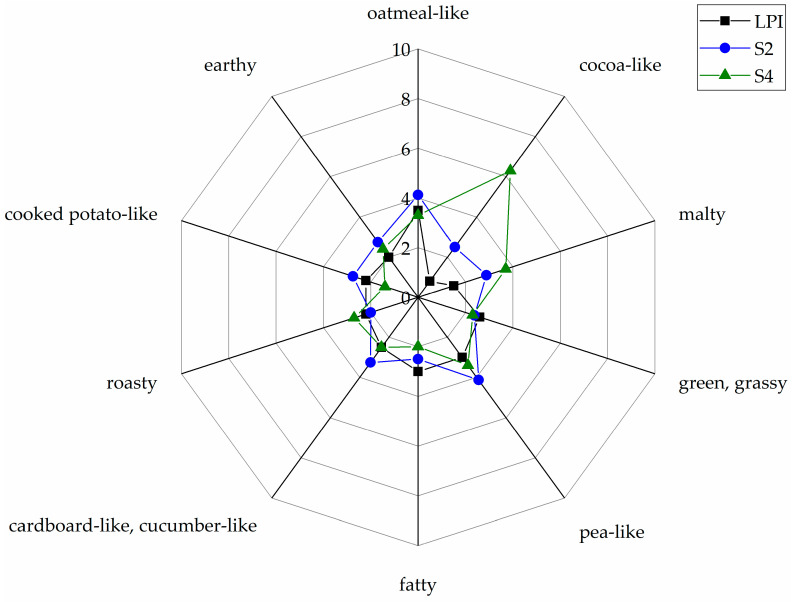
Comparative retronasal aroma profile analyses of LPI and fermented LPI hydrolysates (S2 and S4) on a scale from 0 (no perception) to 10 (strong perception). The data are displayed as mean values of the sensory evaluation (n = 10).

Comparative retronasal aroma profile analysis (Figure 4) shows the results of Papain hydrolysate S2 (*Lactobacillus amylolyticus*) and Alcalase 2.4 L hydrolysate S4 (*Lactobacillus sakei* ssp. *carnosus*) compared to LPI across the panel and highlights the most impressive changes with significant differences (*p* < 0.05) in aroma perception of cocoa-like and malty. For LPI, the major aroma perceptions were rated with a moderate perception with values of 3.5 for oatmeal-like, 3.0 for fatty and 3.0 for pea-like. All other aroma impressions were rated with an intensity of 2.6 and below. The Papain hydrolysate S2 (*Lactobacillus amylolyticus*) and the Alcalase 2.4 L hydrolysate S4 (*Lactobacillus sakei* ssp*. carnosus*) were the only samples that showed significant differences (*p* < 0.05) in the intensity of aroma perception (cocoa-like and malty) compared to untreated LPI. The cocoa-like impression increased with values of 2.5 for S2 and 6.3 for S4 compared to 0.8 for unmodified LPI. Furthermore, S4 showed a significant increase (*p* < 0.05) in the intensity of the aroma perception of malty (3.7). All other samples showed no significant differences (*p* < 0.05) of aroma perceptions compared to those of unmodified LPI, and are therefore not presented in the figure (see Supplementary Materials).

The taste impressions bitter, salty and sour of LPI and fermented LPI hydrolysates were investigated by 10 panelists in the sensory evaluation. The bitter, salty and sour intensities of LPI were described with values of 3.0, 2.1 and 1.0, respectively. All treated samples did not show significant difference (*p* < 0.05) compared to untreated LPI, with the exception of the sample treated with Alcalase 2.4 L hydrolysate S5 (*Lactobacillus amylolyticus*), with a more bitter intensity of 5.6. Additionally, Pepsin hydrolysate S9 (*Lactobacillus helveticus*) showed significantly (*p* < 0.05) higher intensity of saltiness compared to untreated LPI. No significant differences could be observed between LPI and the treated samples regarding the intensity of sour.

**Figure 5:**
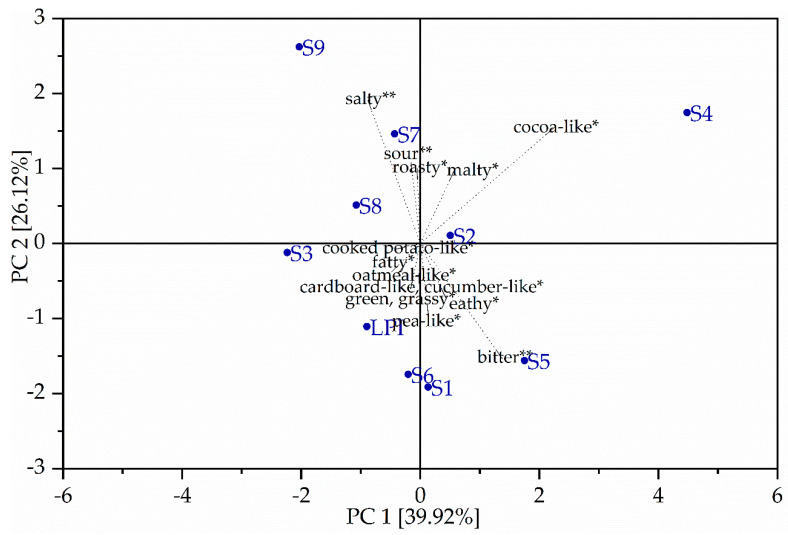
Biplot of aroma and taste of the unfermented LPI and fermented LPI hydrolysates. * aroma ** taste.

Principal Component Analysis (PCA) was applied to identify the most relevant sensory attributes that affect sample characteristics. Figure 5 shows the resulting biplots of the uncorrelated principal components (PCs) 1 and 2 based on the sensory data of the respective hydrolysates including untreated LPI, fermented LPI hydrolysates and the scaled loadings.

The first two components of PCA explained 39.92% and 26.12% of the observed variation (68.22% in total). The attribute with the strongest influence on PC1 was salty (−0.298) taste and cocoa-like (0.754) aroma impression. In contrast, PC2 was primarily described by the attributes bitter (−0.412) and salty (0.514). Unfermented LPI (−0.896/−1.108) was in close correlation with the pea-like aroma attribute and was found on the negative side of PC2, together with the Papain hydrolysates samples S1 (*Lactobacillus sakei* ssp*. carnosus*) and S3 (*Lactobacillus helveticus*) and the Alcalase 2.4 L hydrolysates samples S5 (*Lactobacillus amylolyticus*) and S6 (*Lactobacillus helveticus*). The samples S2, S4 and the Pepsin hydrolysates S7 (*Lactobacillus sakei* ssp*. carnosus*), S8 (*Lactobacillus amylolyticus*) and S9 (*Lactobacillus helveticus*) were found on the positive side and were opposite of the unfermented LPI. The Pepsin hydrolysate fermented with *L. helveticus* (S9) scored the highest in the PC2 (2.623) and was nearest with salty. S9 raised a low pH value of 3.7 after fermentation, which was neutralized (pH 7.0) with 1M NaOH. Due to the larger amount of NaOH, compared to other samples, the salty impression may have been increased. This assumption is further supported by the increased ash content for S9. In contrast, the Papain hydrolysate S1 (*Lactobacillus sakei* ssp*. carnosus*) and the Alcalase 2.4 L hydrolysates S5 (*Lactobacillus amylolyticus*) and S6 (*Lactobacillus helveticus*) scored the lowest in PC2 and were nearest ranged to the bitter taste attribute. It was observed that the Alcalase 2.4 L hydrolysate S5 (*Lactobacillus amylolyticus*) showed a significant difference (*p* < 0.05) of bitter intensity to untreated LPI. The Alcalase 2.4 L hydrolysates S4 (*Lactobacillus sakei* ssp*. carnosus*) and S6 (*Lactobacillus helveticus*) did not show a significant difference (*p* < 0.05), but a tendency of higher bitter intensity. Alcalase 2.4 L hydrolysates are known for bitter taste. Several studies have already shown that the hydrolysis of plant proteins such as lupin [7,10], soy [6] and pea [27] results in a bitter taste. However, S4 (*Lactobacillus sakei* ssp*. carnosus*) was not ranged close to the bitter attribute, rather in the positive range of PC2 (1.746), and was nearest ranged with cocoa-like and was scored the highest in the PC1 (4.483). 

## 4. Conclusions

The combination of enzymatic hydrolysis and fermentation of lupin protein isolate can increase foaming activity while maintaining proper emulsification capacity. In addition, the modification increases protein solubility at acidic conditions and thus opens new possibilities in food applications such as refreshing drinks. LPI provides a well-balanced sensory profile that has been partially altered by the treatments in aroma and taste perception. The sensory acceptance of LPI and modified LPI needs to be investigated with a consumer panel. SDS-PAGE and Bead-Assay indicated that the combination of enzymatic hydrolysis and fermentation of LPI is effective in breaking down large polypeptides into low molecular weight peptides and degrading with it the major allergen Lup an 1 of *L. angustifolius*. Thus, this two-step process represents a promising method for the reduction of the allergenic potential of LPI. Nevertheless, in vivo studies should be performed to investigate the allergenicity of fermented lupin protein hydrolysates. The use and possible applications of fermented lupin protein hydrolysates in food should be tested in practice by subsequent application trails.

## Figures and Tables

**Table 1:** Sources, types and supplier of the enzymes used in this study.

Enzyme	Type	Biological Source	Supplier
Papain	cysteine endopeptidase	Papaya (*Carica* sp.) latex	AppliChem GmbH (Darmstadt, Germany)
Alcalase 2.4 L FG	serine endopeptidase	*Bacillus licheniformis*	Novozymes A/S (Bagsvaerd, Denmark)
Pepsin	aspartic endopeptidase	Porcine (Sus domesticus) gastric mucosa	Merck KGaA(Darmstadt, Germany)

**Table 2:** Experimental design of enzymatic hydrolysis and fermentation with enzyme-to-solution ratio (E/S), temperature and pH value of enzymatic hydrolysis.

System		E/S (%) ^1^	Temperature (°C)	pH Value (-)
**Papain**		0.2	80	7.0
S1	*Lactobacillus sakei* ssp. *carnosus*			
S2	*Lactobacillus amylolyticus*			
S3	*Lactobacillus helveticus*			
**Alcalase 2.4 L**		0.5	50	8.0
S4	*Lactobacillus sakei* ssp. *carnosus*			
S5	*Lactobacillus amylolyticus*			
S6	*Lactobacillus helveticus*			
**Pepsin**		0.5	50	2.0
S7	*Lactobacillus sakei* ssp. *carnosus*			
S8	*Lactobacillus amylolyticus*			
S9	*Lactobacillus helveticus*			

^1^ enzyme-to-solution ratio of enzymatic hydrolysis.

**Table 3:** Dry matter (%), protein content (%) and ash content (%) of lupin protein isolate (LPI) and fermented LPI hydrolysates.

Samples	Dry Matter (%)	Protein Content (%)	Ash Content (%)
LPI	95.4 ± 0.0	89.6 ± 0.0	4.2 ± 0.12
Papain			
S1	93.8 ± 0.0 *	74.7 ± 2.5	6.7 ± 0.9
S2	93.6 ± 0.2 *	78.7 ± 2.0	5.4 ± 0.6
S3	93.1 ± 0.0 *	66.8 ± 0.0 *	6.5 ± 1.1
Alcalase 2.4 L			
S4	92.8 ± 0.2 *	74.8 ± 0.6 *	6.2 ± 0.9 *
S5	93.5 ± 0.3 *	75.6 ± 1.3 *	5.3 ± 0.4
S6	93.0 ± 0.2 *	77.2 ± 5.6	7.4 ± 0.1 *
Pepsin			
S7	94.0 ± 0.0 *	73.1 ± 5.9	7.1 ± 0.4
S8	93.9 ± 0.1 *	78.0 ± 1.5	6.3 ± 0.6
S9	93.7 ± 0.2 *	72.2 ± 4.5	8.8 ± 0.7 *

The data are expressed as mean ± standard deviation (*n* = 4). Means marked with an asterisk (*) within a column indicate significant differences between the sample and the untreated LPI (*p* < 0.05) following pairwise *t*-test.

**Table 4:** Colony forming units (CFU) after 0 h and 24 h of fermentation.

Samples	CFU/mL		
	0 h	24 h	ΔE_CFU/mL_
Papain			
S1	2.25 × 10^7^ ± 2.12 × 10^6^	4.86 × 10^8^ ± 7.85 × 10^7^	4.63 × 10^8^ ± 8.06 × 10^7^
S2	1.03 × 10^7^ ± 1.20 × 10^6^	1.03 × 10^9^ ± 1.41 × 10^7^	1.02 × 10^9^ ± 1.29 × 10^7^
S3	6.45 × 10^6^ ± 1.30 × 10^6^	2.10 × 10^8^ ± 5.21 × 10^7^	2.03 × 10^8^ ± 5.09 × 10^7^
Alcalase 2.4 L			
S4	1.26 × 10^7^ ± 1.91 × 10^6^	1.30 × 10^9^ ± 7.21 × 10^8^	1.29 × 10^9^ ± 7.23 × 10^8^
S5	1.36 × 10^7^ ± 1.41 × 10^6^	1.23 × 10^9^ ± 3.56 × 10^8^	1.21 × 10^9^ ± 3.54 × 10^8^
S6	8.45 × 10^6^ ± 1.53 × 10^6^	3.62 × 10^8^ ± 1.63 × 10^8^	3.54 × 10^8^ ± 1.61 × 10^8^
Pepsin			
S7	7.01 × 10^6^ ± 1.34 × 10^5^	1.97 × 10^8^ ± 1.91 × 10^7^	1.89 × 10^8^ ± 1.90 × 10^7^
S8	1.14 × 10^7^ ± 2.88 × 10^6^	1.34 × 10^9^ ± 2.62 × 10^8^	1.32 × 10^9^ ± 2.59 × 10^8^
S9	1.06 × 10^7^ ± 2.12 × 10^6^	1.12 × 10^8^ ± 6.48 × 10^7^	1.02 × 10^8^ ± 6.70 × 10^7^

The data are expressed as mean ± standard deviations from duplicates.

**Table 5:** pH values after 0 h and 24 h of fermentation.

Samples	pH-Values	
	0 h	24 h
Papain		
S1	7.1 ± 0.1	4.6 ± 0.1
S2	7.1 ± 0.0	4.9 ± 0.1
S3	6.9 ± 0.3	3.3 ± 0.2
Alcalase 2.4 L		
S4	7.1 ± 0.0	5.1 ± 0.0
S5	7.0 ± 0.1	4.8 ± 0.1
S6	7.3 ± 0.3	4.9 ± 0.4
Pepsin		
S7	7.0 ± 0.0	4.9 ± 0.1
S8	7.1 ± 0.0	4.9 ± 0.0
S9	7.3 ± 0.2	3.7 ± 0.3

The data are expressed as mean ± standard deviations from duplicates.

**Table 6:** Protein solubility, foam properties and emulsifying capacity of LPI and fermented LPI hydrolysates.

Samples	Protein Solubility		Foaming Activity		Foam Stability		Emulsifying Capacity	
	pH 4.0	pH 7.0	pH 4.0	pH 7.0	pH 4.0	pH 7.0	pH 4.0	pH 7.0
	(%)	(%)	(%)	(%)	(%)	(%)	(%)	(%)
LPI	7.3 ± 0.3	63.6 ± 3.0	828 ± 3	1613 ± 11	92 ± 1	89 ± 0	410 ± 7	666 ± 0
Papain								
S1	24.8 ± 3.2 *	53.3 ± 7.7	2118 ± 47 *	2544 ± 39 *	0 ± 0 *	1 ± 0 *	350 ± 21	393 ± 15 *
S2	19.7 ± 2.4 *	45.2 ± 10.7	1819 ± 38 *	2606 ± 53 *	0 ± 0 *	38 ± 8 *	340 ± 0 *	432 ± 65 *
S3	36.7 ± 3.0 *	66.6 ± 6.9	2395 ± 45 *	2505 ± 54 *	0 ± 0 *	29 ± 6 *	415 ± 28	432 ± 9 *
Alcalase 2.4 L								
S4	23.4 ± 4.6 *	60.4 ± 9.3	2458 ± 58 *	2466 ± 54 *	0 ± 0 *	96 ± 3	358 ± 32	246 ± 28 *
S5	25.6 ± 8.8 *	55.0 ± 7.9	2766 ± 54 *	2676 ± 56 *	0 ± 0 *	96 ± 2 *	258 ± 18	283 ± 30 *
S6	27.3 ± 1.7 *	46.0 ± 3.8 *	2789 ± 28 *	2721 ± 91	0 ± 0 *	0 ± 0 *	429 ± 8 *	323 ± 3 *
Pepsin								
S7	25.7 ± 5.0 *	57.5 ± 12.2	1819 ± 51 *	3338 ± 71 *	0 ± 0 *	41 ± 1 *	400 ± 35	477 ± 16 *
S8	24.0 ± 2.1 *	56.8 ± 6.3	1993 ± 46 *	3481 ± 39 *	0 ± 0 *	90 ± 4	400 ± 7 *	439 ± 5 *
S9	25.6 ± 2.7 *	55.7 ± 11.9	2001 ± 40 *	3443 ± 51 *	0 ± 0 *	7 ± 1 *	405 ± 0	417 ± 6 *

The data are expressed as mean ± standard deviation (*n* = 4). Means marked with an asterisk (*) within a column indicate significant differences between sample and untreated LPI (*p* < 0.05) following pairwise *t*-test.

## Supplementary Materials

The following are available online at https://www.mdpi.com/2304-8158/10/2/281/s1, Table S1: Comparative retronasal aroma profile analyses of LPI and fermented LPI hydrolysates, Table S2: Intensity of bitter, salty and sour taste perception of LPI and fermented LPI hydrolysates, Table S3: Principal Component (PC) scaled scores and loadings for LPI and fermented LPI hydrolysates obtained by Principal Component Analysis (PCA).

## References

[1] (2020). Word Population Prospects—The 2019 Revision.

[2] Eshel G., Shepon A., Makov T., Milo R. (2014). Land, irrigation water, greenhouse gas, and reactive nitrogen burdens of meat, eggs, and dairy production in the United States. Proc. Natl. Acad. Sci. USA.

[3] Bähr M., Fechner A., Hasenkopf K., Mittermaier S., Jahreis G. (2014). Chemical composition of dehulled seeds of selected lupin cultivars in comparison to pea and soya bean. LWT—Food Sci. Technol..

[4] Bader S., Oviedo J.P., Pickardt C., Eisner P. (2011). Influence of different organic solvents on the functional and sensory properties of lupin (*Lupinus angustifolius* L.) proteins. LWT—Food Sci. Technol..

[5] Purschke B., Meinlschmidt P., Horn C., Rieder O., Jäger H. (2018). Improvement of techno-functional properties of edible insect protein from migratory locust by enzymatic hydrolysis. Eur. Food Res. Technol..

[6] Meinlschmidt P., Sussmann D., Schweiggert-Weisz U., Eisner P. (2016). Enzymatic treatment of soy protein isolates: Effects on the potential allergenicity, technofunctionality, and sensory properties. Food Sci. Nutr..

[7] Schlegel K., Sontheimer K., Hickisch A., Wani A.A., Eisner P., Schweiggert-Weisz U. (2019). Enzymatic hydrolysis of lupin protein isolates—Changes in the molecular weight distribution, technofunctional characteristics and sensory attributes. Food Sci. Nutr..

[8] Akbari N., Mohammadzadeh Milani J., Biparva P. (2020). Functional and conformational properties of proteolytic enzyme-modified potato protein isolate. J. Sci. Food Agric..

[9] Klost M., Drusch S. (2019). Functionalisation of pea protein by tryptic hydrolysis—Characterisation of interfacial and functional properties. Food Hydrocoll..

[10] Schlegel K., Sontheimer K., Eisner P., Schweiggert-Weisz U. (2019). Effect of enzyme-assisted hydrolysis on protein pattern, technofunctional, and sensory properties of lupin protein isolates using enzyme combinations. Food Sci. Nutri.

[11] Meng S., Tan Y., Chang S., Li J., Maleki S., Puppala N. (2020). Peanut allergen reduction and functional property improvement by means of enzymatic hydrolysis and transglutaminase crosslinking. Food Chem..

[12] Lqari H., Pedroche J., Girón-Calle J., Vioque J., Millán F. (2005). Production of *Lupinus angustifolius* protein hydrolysates with improved functional properties. Grasas y Aceites.

[13] Schindler S., Wittig M., Zelena K., Krings U., Bez J., Eisner P., Berger R.G. (2011). Lactic fermentation to improve the aroma of protein extracts of sweet lupin (Lupinus angustifolius). Food Chem..

[14] Schindler S., Zelena K., Krings U., Bez J., Eisner P., Berger R.G. (2012). Improvement of the Aroma of Pea (Pisum sativum) Protein Extracts by Lactic Acid Fermentation. Food Biotechnol..

[15] Schlegel K., Leidigkeit A., Eisner P., Schweiggert-Weisz U. (2019). Technofunctional and Sensory Properties of Fermented Lupin Protein Isolates. Foods.

[16] Mosse J., Huet J.-C., Baudet J. (1987). Relationships between nitrogen, amino acids and storage proteins in *Lupinus albus* seeds. Phytochemistry.

[17] Laemmli U.K. (1970). Cleavage of structural proteins during the assembly of the head of bacteriophage T4. Nature.

[18] Morr C.V., German B., Kinsella J.E., Regenstein J.M., Vanburen J.P., Kilara A., Lewis B.A., Mangino M.E. (1985). A Collaborative Study to Develop a Standardized Food Protein Solubility Procedure. J. Food Sci..

[19] Lowry O.H., Rosebrough N.J., Farr A.L., Randall R.J. (1951). Protein measurement with the Folin phenol reagent. J. Biol. Chem..

[20] Phillips L.G., Haque Z., Kinsella J.E. (1987). A Method for the Measurement of Foam Formation and Stability. J. Food Sci..

[21] Wang C.Y., Johnson L.A. (2001). Functional properties of hydrothermally cooked soy protein products. J. Am. Oil Chem. Soc..

[22] Goggin D.E., Mir G., Smith W.B., Stuckey M., Smith P.A.C. (2008). Proteomic analysis of lupin seed proteins to identify conglutin beta as an allergen, Lup an 1. J. Agric. Food Chem..

[23] Sormus de Castro Pinto S.E., Neves V.A., Machado de Medeiros B.M. (2009). Enzymatic Hydrolysis of Sweet Lupin, Chickpea, and Lentil 11S Globulins Decreases their Antigenic Activity. J. Agric. Food Chem..

[24] Huang T., Bu G., Chen F. (2018). The influence of composite enzymatic hydrolysis on the antigenicity of β-conglycinin in soy protein hydrolysates. J. Food Biochem..

[25] Kasera R., Singh A.B., Lavasa S., Prasad K.N., Arora N. (2015). Enzymatic hydrolysis: A method in alleviating legume allergenicity. Food Chem. Toxicol..

[26] Meinlschmidt P., Schweiggert-Weisz U., Eisner P. (2016). Soy protein hydrolysates fermentation: Effect of debittering and degradation of major soy allergens. LWT—Food Sci. Technol..

[27] Garcia-Arteaga V., Apestegui M., Muranyi I., Eisner P., Schweiggert-Weisz U. (2020). Effect of enzymatic hydrolysis on molecular weight distribution, techno-functional properties and sensory perception of pea protein isolates. Innov. Food Sci. Emerg. Technol..

[28] Muranyi I.S., Otto C., Pickardt C., Osen R., Koehler P., Schweiggert-Weisz U. (2016). Influence of the Isolation Method on the Technofunctional Properties of Protein Isolates from Lupinus angustifolius L.. J. Food Sci..

[29] Vogelsang-O’Dwyer M., Bez J., Petersen I., Joehnke M., Detzel A., Busch M., Krueger M., Ispiryan L., O’Mahony J., Arendt E. (2020). Techno-Functional, Nutritional and Environmental Performance of Protein Isolates from Blue Lupin and White Lupin. Foods.

[30] Klupsaite D., Juodeikiene G., Zadeike D., Bartkiene E., Maknickiene Z., Liutkute G. (2017). The influence of lactic acid fermentation on functional properties of narrow-leaved lupine protein as functional additive for higher value wheat bread. LWT—Food Sci. Technol..

[31] Tsumura K., Saito T., Tsuge K., Ashida H., Kugimiya W., Inouye K. (2005). Functional properties of soy protein hydrolysates obtained by selective proteolysis. LWT—Food Sci. Technol..

[32] El-Adawy T.A., Rahma E.H., El-Bedawey A.A., Gafar A.F. (2001). Nutritional potential and functional properties of sweet and bitter lupin seed protein isolates. Food Chem..

