# Supplementary material for: Fermentation of Lupin Protein Hydrolysates—Effects on Their Functional Properties, Sensory Profile and the Allergenic Potential of the Major Lupin Allergen Lup an 1
Source: Foods. 2021 Jan 31;10(2):281. doi: 10.3390/foods10020281 (PMC7910967; doi:10.3390/foods10020281)
Supplement: Supplementary file 1 [file foods-10-00281-s001.zip › Table S1.docx]

Table S1. Comparative retronasal aroma profile analyses of LPI and fermented LPI hydrolysates.

| Samples | oatmeal-like | cocoa-like | malty | green, grassy | pea-like | fatty | cardboard-like, cucumber-like | roasty | cooked potato-like | earthy |
| --- | --- | --- | --- | --- | --- | --- | --- | --- | --- | --- |
| LPI | 3.5 ± 3.0 | 0.8 ± 1.3 | 1.5± 1.5 | 2.6 ± 2.2 | 3.0 ± 2.5 | 3.0 ± 1.8 | 2.5 ± 2.2 | 2.2 ± 1.7 | 2.2 ± 1.9 | 2.0 ± 1.9 |
| Papain |  |  |  |  |  |  |  |  |  |  |
| S1 | 5.5 ± 1.8 | 1.4 ± 1.3 | 2.9 ± 2.4 | 3.0 ± 1.9 | 3.8 ± 2.9 | 2.3 ± 1.7 | 3.9 ± 2.9 | 1.7 ± 1.8 | 2.6 ± 2.5 | 2.6 ± 2.7 |
| S2 | 4.1 ± 2.4 | 2.5 ± 2.1* | 2.9 ± 2.2 | 2.4 ± 1.7 | 4.1 ± 2.9 | 2.5 ± 1.5 | 3.3 ± 2.8 | 2.0 ± 1.6 | 2.8 ± 1.8 | 2.8 ± 1.6 |
| S3 | 4.8 ± 1.7 | 0.6 ± 0.9 | 1.9 ± 1.1 | 2.9 ± 2.1 | 4.1 ± 2.3 | 3.4 ± 2.4 | 2.4 ± 2.1 | 2.4 ± 1.5 | 2.4 ± 1.8 | 1.9 ± 1.1 |
| Alcalase 2.4 L |  |  |  |  |  |  |  |  |  |  |
| S4 | 3.3 ± 2.3 | 6.3 ± 1.5* | 3.7 ± 1.8* | 2.3 ± 2.5 | 3.4 ± 2.1 | 2.0 ± 1.8 | 2.5 ± 1.3 | 2.7 ± 2.0 | 1.4 ± 1.6 | 2.4 ± 2.5 |
| S5 | 3.2 ± 2.3 | 2.5 ± 2.7 | 1.8 ± 1.4 | 3.2 ± 2.0 | 4.1 ± 2.2 | 2.3 ± 2.2 | 2.5 ± 1.5 | 2.3 ± 1.9 | 2.4 ± 1.4 | 3.1 ± 1.7 |
| S6 | 3.2 ± 1.9 | 1.2 ± 1.8 | 1.5 ± 1.6 | 3.8 ± 2.3 | 4.6 ± 2.2 | 3.3 ± 1.7 | 3.3 ± 1.9 | 1.4 ± 1.3 | 2.3 ± 2.2 | 3.0 ± 2.5 |
| Pepsin |  |  |  |  |  |  |  |  |  |  |
| S7 | 3.5 ± 2.5 | 1.9 ± 2.3 | 2.3 ± 1.3 | 2.2 ± 1.5 | 2.8 ± 1.8 | 2.2 ± 2.3 | 2.2 ± 1.9 | 3.0 ± 1.4 | 2.3 ± 1.6 | 2.0 ± 2.2 |
| S8 | 3.8 ± 3.0 | 1.3 ± 1.6 | 2.1 ± 1.1 | 2.4 ± 1.9 | 3.3 ± 2.0 | 3.0 ± 2.3 | 2.8 ± 1.8 | 3.3 ± 1.8 | 2.1 ± 1.7 | 1.5 ± 1.6 |
| S9 | 3.8 ± 2.3 | 1.6 ± 2.1 | 2.9 ± 1.8 | 3.1 ± 2.2 | 2.9 ± 2.2 | 2.5 ± 1.8 | 2.9 ± 2.8 | 2.8 ± 2.5 | 3.0 ± 1.6 | 1.9 ± 2.1 |

The data are expressed as mean ± standard deviation (n = 10) on a scale from 0 (no perception) to 10 (strong perception). Means marked with an asterisk (*) within a column indicate significant differences between sample and untreated LPI (p < 0.05) following pairwise t-test.
