# Supplementary material for: Fermentation of Lupin Protein Hydrolysates—Effects on Their Functional Properties, Sensory Profile and the Allergenic Potential of the Major Lupin Allergen Lup an 1
Source: Foods. 2021 Jan 31;10(2):281. doi: 10.3390/foods10020281 (PMC7910967; doi:10.3390/foods10020281)
Supplement: Supplementary file 1 [file foods-10-00281-s001.zip › Table S2.docx]

Table S2. Intensity of bitter, salty and sour taste perception of LPI and fermented LPI hydrolysates.

| Samples | bitter | salty | sour |
| --- | --- | --- | --- |
| LPI | 3.0 ± 1.6 | 2.1 ± 1.9 | 1.0± 0.8 |
| Papain |  |  |  |
| S1 | 3.3 ± 2.5 | 1.4 ± 1.1 | 1.0 ± 0.9 |
| S2 | 2.9 ± 2.2 | 2.4 ± 1.8 | 2.9 ± 2.6 |
| S3 | 1.9 ± 1.1 | 3.9 ± 1.2 | 1.0 ± 0.9 |
| Alcalase 2.4 L |  |  |  |
| S4 | 4.3 ± 1.8 | 2.6 ± 1.8 | 1.9 ± 1.6 |
| S5 | 5.6 ± 2.5* | 2.8 ± 2.0 | 0.8 ± 0.8 |
| S6 | 4.8 ± 2.3 | 4.0 ± 2.6 | 1.5 ± 1.1 |
| Pepsin |  |  |  |
| S7 | 3.2 ± 1.5 | 4.3 ± 2.7 | 2.4 ± 2.2 |
| S8 | 2.5 ± 1.5 | 3.1 ± 1.7 | 1.9 ± 1.8 |
| S9 | 1.4 ± 1.0 | 5.8 ± 1.0* | 2.5 ± 2.3 |

The data are expressed as mean ± standard deviation (n = 10) on a scale from 0 (no perception) to 10 (strong perception). Means marked with an asterisk (*) within a column indicate significant differences between sample and untreated LPI (p < 0.05) following pairwise t-test.
