# Supplementary material for: Fermentation of Lupin Protein Hydrolysates—Effects on Their Functional Properties, Sensory Profile and the Allergenic Potential of the Major Lupin Allergen Lup an 1
Source: Foods. 2021 Jan 31;10(2):281. doi: 10.3390/foods10020281 (PMC7910967; doi:10.3390/foods10020281)
Supplement: Supplementary file 1 [file foods-10-00281-s001.zip › Table S3.docx]

Table S3. Principal Component (PC) scaled scores and loadings for LPI and fermented LPI hydrolysates obtained by Principal Component Analysis (PCA).

| Scaled scores | | | Scaled loadings | | |
| --- | --- | --- | --- | --- | --- |
| Samples | PC1 | PC2 | Attributes | PC1 | PC2 |
| LPI | -0,896 | -1,108 | oatmeal-like* | -0,127 | -0,108 |
| Papain |  |  | cocoa-like* | 0,754 | 0,413 |
| S1 | 0,132 | -1,914 | malty* | 0,191 | 0,261 |
| S2 | 0,506 | 0,106 | green, grassy* | -0,054 | -0,162 |
| S3 | -2,235 | -0,123 | pea-like* | 0,045 | -0,252 |
| Alcalase 2.4 L |  |  | fatty* | -0,161 | -0,102 |
| S4 | 4,483 | 1,746 | cardboard-like, cucumber-like* | -0,008 | -0,142 |
| S5 | 1,751 | -1,561 | roasty* | -0,018 | 0,269 |
| S6 | -0,203 | -1,744 | cooked potato-like* | -0,140 | -0,021 |
| Pepsin |  |  | earthy* | 0,145 | -0,196 |
| S7 | -0,431 | 1,462 | bitter** | 0,465 | -0,412 |
| S8 | -1,075 | 0,513 | salty** | -0,297 | 0,514 |
| S9 | -2,033 | 2,623 | sour** | -0,052 | 0,290 |

*aroma, **taste
